# The Effects of 24 Weeks Sensorimotor Training on Balance, Physical Function, and Pain in Women with Knee Osteoarthritis

**DOI:** 10.3390/sports14010043

**Published:** 2026-01-16

**Authors:** Caterina Mauri, Charles James Steward, Attilio Parisi, Mathew Hill, Sara Severoni, Claudia Cerulli, Elisa Grazioli

**Affiliations:** 1 Unit of Physical Exercise and Sport Sciences, Department of Movement, Human and Health Sciences, University of Rome «Foro Italico», 00135 Rome, Italy; c.mauri@studenti.uniroma4.it (C.M.); claudia.cerulli@uniroma4.it (C.C.); elisa.grazioli@uniroma4.it (E.G.); 2 School of Life Sciences, University of Nottingham, Nottingham NG7 2RD, UK; charles.steward@nottingham.ac.uk; 3 School of Psychology and Vision Sciences, University of Leicester, Leicester LE1 7RH, UK; mwh20@leicester.ac.uk; 4 Associazione Laziale Malati Reumatici aps (ALMAR aps), Ospedale San Camillo Forlanini, 00152 Rome, Italy; saseveroni@gmail.com

**Keywords:** Rheumatic diseases, exercise, postural sway, pain, functional abilities, quality of life

## Abstract

Background: Osteoarthritis (OA) is a common degenerative joint disease that often leads to impaired postural control, pain, and reduced physical function. Exercise is considered a first-line treatment, with sensorimotor training being an effective approach for managing OA. However, the optimal method of sensorimotor training for individuals with OA has not yet been established. Thus, the aim of this study was to compare the effects of a 24-week Gyrokinesis method (GK) versus Pilates (PL) intervention on balance control, function, pain and kinesiophobia in women with knee OA. Methods: Twenty women (aged 60 ± 7 years) with grade 2 or 3 knee OA were assigned to either GK (*n* = 12) or PL (*n* = 8). Both groups trained twice weekly for 24 weeks. Pre- and post-intervention assessments included postural sway parameters (RMS, velocity, frequency), physical function tests (e.g., TUG, Sit-to-Stand), flexibility, pain (Brief Pain Inventory), kinesiophobia (Tampa Scale), and quality of life (SF-36). Results: GK resulted in significantly greater improvements than PL in postural sway mean velocity AP right (GK −53.85% vs. PL −20.17%), AP left (GK −43.48% vs. PL +13.45%), and ML left (GK −40.18% vs. PL +37.95), pain reduction (GK −82.5% vs. PL −33.3%), and physical function (Sit-to-Stand: GK +75.9% vs. PL +3.7%; TUG: GK −16.4% vs. PL −13.8%; Step Test right: GK +34.2% vs. PL +19.9%; Step Test left: GK +41.4% vs. PL +18.1%) (all, *p* < 0.05). No significant between-group differences were observed for kinesiophobia or SF-36 scores (both, *p* > 0.05). Conclusions: Gyrokinesis method may be more effective than Pilates in enhancing balance, reducing pain, and improving physical function in women with knee OA. These findings support the use of the Gyrokinesis method in rehabilitation programs for individuals with OA.

## 1. Introduction

Osteoarthritis (OA) is a degenerative joint disorder that affects ~8% of the global population [1] and the 15th leading cause of years lived with disabilities worldwide [2,3]. Over the past 30 years, the number of OA diagnoses has increased by ~132% [4], with the knee joint being particularly susceptible [1,5]. The pathophysiology of OA is characterised by the degradation of joint structures caused by a combination of mechanical stress and inflammation [2]. This can lead to symptoms including pain, stiffness, and swelling [6,7]. Collectively, these symptoms limit function and mobility, disrupt daily activities, and negatively impact personal, social, and psychological well-being [7].

Individuals with knee OA often exhibit impaired balance control, characterised by increased postural sway, along with quadriceps weakness and reduced proprioception compared to age- and sex-matched controls [8]. These deficits are particularly evident in those experiencing higher levels of pain, who tend to show greater functional limitations and postural instability than asymptomatic individuals [9]. While pain and muscle strength are believed to influence balance control, further interventional research is needed to clarify these underlying mechanisms. Accordingly, promoting regular physical activity is likely crucial for improving clinical outcomes and mitigating fall risk in this population.

Pain is often identified as the most debilitating symptom of OA, triggering a negative cycle of muscle disuse, joint stiffness and reduced range of motion, which together further compromise balance and increase the risk of falls [10]. Exercise has emerged as a first-line, low-risk, and cost-effective strategy for reducing pain and improving physical function, balance and overall quality of life in individuals with OA [11,12,13]. Both aerobic and strength exercises have been demonstrated to be effective in the management of OA symptoms [14]. However, a recent systematic review and meta-analysis suggests that neuromuscular or sensorimotor training, may be particularly effective in managing pain and improving physical function in individuals with OA [15,16]. Among these, Pilates (PL) is a widely practiced training approach that focuses on joint stability, posture, balance, and controlled movement [17]. Indeed, evidence suggests that as little as 8 weeks of Pilates training can significantly reduce pain and improve physical function and balance in patients with OA [17,18].

Another emerging sensorimotor approach is the Gyrokinesis method (GK), which incorporates three-dimensional and spiral movements that integrate elements of yoga, dance and tai chi [19]. Similarly to PL, GK also targets joint mobility, spinal articulation, postural control, and functional movement patterns and therefore has the potential to offer similar benefits to individuals with musculoskeletal disorders [19]. Although research into Gyrokinesis is still in its early stages, preliminary studies have reported improvements in gait parameters (e.g., step length, stride length, gait speed) and lumbar stability after 4–8 weeks of training in individuals with low back pain [20,21]. Therefore, the primary aim of this exploratory study was to investigate the effect of a 24-week Gyrokinesis and Pilates intervention on balance control, functional parameters, pain, and kinesiophobia in individuals with knee OA. Additionally, we aimed to compare the outcomes between these two sensorimotor training modalities to evaluate their relative efficacy in alleviating OA-related symptoms.

## 2. Materials and Methods

### 2.1. Ethical Approval

The study was approved by the ethical committee of the Lazio 1- San Camillo Hospital, Rome, Italy (Prot n 330/CE LAZIO 1 of 12 April 2023). All experimental procedures conformed to the Declaration of Helsinki, with the exception of prior registration in a public database [22]. All participants provided written informed consent before enrolment in the study.

### 2.2. Participants

Twenty female participants with knee osteoarthritis (KOA) were enrolled (age: 60 ± 7 years; weight: 68 ± 10 kg; height: 165 ± 6 cm; body mass index: 25 ± 3 kg/m^2^). Participants were aged between 45–70 years and took part in less than 50 min of moderate intensity exercise per week. All participants were diagnosed with grade 2 or 3 OA according to the Kellgren/Lawrence classification (KL grade 2 *n* = 5; KL grade 3 *n* = 15) [23] and reported a Brief Pain Inventory (BPI) [24] score greater than 1.5. Exclusion criteria included other forms of arthritis, comorbidities, recent knee arthroplasty, and injuries. Participants on painkillers or anti-inflammatory drugs were required to maintain a consistent dosage throughout the study. Only participants who attended at least 80% of the scheduled exercise sessions were included in the analysis.

### 2.3. Experimental Design

Participants were allocated non-randomly (based on geographical location) to either 24 weeks of GK (*n* = 12) or Pilates (*n* = 8). Over the 24 weeks, participants attended supervised sessions twice weekly (48 total sessions), in small groups (4–5 participants) at either 1–2 pm or 4–5 pm. Outcome measures were assessed before and after the intervention, with all assessments conducted between 8:00–10:00 to control for the effects of circadian rhythm on outcome measures.

### 2.4. Intervention Characteristics

#### 2.4.1. Gyrokinesis Method Training

Sessions were conducted by a licensed Level 1 trainer certified by the Gyrokinesis International Headquarters and followed the core principles of the method: synchronizing with corresponding breathing patterns, stabilization through opposition, joint space creation, scooping, and pelvis narrowing with lumbar spine decompression [20]. Training began with self-administered foot massages and progressed to isometric/dynamic lower limb exercises. Multidirectional spinal movements (arches, curls, spirals, circles) and abdominal engagement exercises (“seed centre connection”) were included. Sessions concluded with balance and proprioceptive tasks. Exercises were repeated 8–16 times and performed on a stool or mat (see Table 1 for full sequence of exercises).

#### 2.4.2. Pilates Training

Conducted by a certified Pilates instructor, sessions involved mat-based exercises in supine and standing positions. The program adhered to six core principles: concentration, centering, control, breathing, flow, and precision [25]. Training included breathing drills, core engagement, spinal mobility, standing balance work, and final flexibility-focused stretches. Unlike Gyrokinesis, Pilates exercises were segmented into discrete sets, with each set consisting of 8–16 repetitions (see Table 1 for full sequence of exercises).

### 2.5. Assessments

#### 2.5.1. Postural Sway

Postural sway was assessed using Microtech’s Gyko inertial sensor (Microgate S.r.l. Bolzano, Italy), positioned at the level of the fifth lumbar vertebra (L5) to the first sacral vertebra (S1). Participants performed a single-leg stance test on a firm surface, barefoot, with eyes open and hands clasped together in front of their body [26]. The test was performed on both the dominant and non-dominant leg, in a randomized order. Each trial lasted a maximum of 30 s. Given postural control is a multifaceted phenomenon, its assessment should include a range of COP descriptors. Non-linear indices, including sample entropy, together with frequency-based measures like mean power frequency, may reveal distinct balance strategies during quiet standing that earlier research has largely overlooked [27]. The validity and reliability of similar parameters have previously been established for this sampling duration [28]. If participants lost balance and touched the ground with the non-stance foot, or used compensatory arm movements, the trial was terminated and the time to failure was recorded. Three trials were performed for each leg, and the best performance (i.e., longest stance time) was used for subsequent analysis.

Body sway parameters were extracted using the manufacturer’s proprietary software (Gyko, Version 1.1.1.10) and included root mean square (RMS), mean velocity (MV), and mean frequency (MF) in both anteroposterior and mediolateral directions. These metrics were chosen to capture distinct dimensions of postural control: RMS reflects sway amplitude/performance, MV quantifies sway speed, and MF estimates the frequency of postural adjustments. Although MV and MF both pertain to the temporal dynamics of sway, they provide complementary insights—MV reflects how rapidly the centre of mass shifts, while MF offers insight into the neuromuscular control strategies, particularly the rate of corrective adjustments. Despite some conceptual overlap, both parameters were retained due to their unique contributions to understanding balance control in individuals with KOA. It is acknowledged that MV may be sensitive to test duration, as longer stance periods may lead to greater cumulative sway. Nonetheless, in populations with impaired balance, where sway tends to increase over time, MV may still capture meaningful differences in dynamic stability. In contrast, MF is considered less sensitive to test duration and more indicative of adaptive postural control mechanisms.

#### 2.5.2. Functional Measures

To assess physical function, flexibility, and range of motion, a battery of seven functional tests recommended by the Osteoarthritis Research Society International (OARSI) [29] was administered. Each test was performed three times, and the best attempt was recorded for analysis. Physical function was evaluated using four standardized tests: the Timed Up and Go (TUG), Stair Climb Test (10 steps), 30-Seconds Sit-to-Stand Test, and the 15-Seconds Step Test [30,31]. Flexibility and range of motion were assessed using the Sit-and-Reach Test (targeting hamstrings and lower back), the Trunk Rotation Test (using the Gyko sensor), and the Back Scratch Test, which evaluates scapulohumeral mobility [32,33].

#### 2.5.3. Perceptual Measures

The Italian version of BPI [24] was used to identify pain location, severity, and the extent to which pain interferes with daily activities, using a 0–10 Likert scale (0 = ‘no pain’ to 10 = ‘the worst pain imaginable’). To assess kinesiophobia, the Italian version of the Tampa Scale for Kinesiophobia (TSK) was used [34]. This 13-item questionnaire evaluates ‘fear of movement’ based on activity avoidance and somatic focus. Quality of life was assessed using the SF-36 Health Survey, which measures self-reported health status across eight domains [35].

#### 2.5.4. Statistical Analysis

Data normality was assessed using the Shapiro–Wilk test. Parametric data were analysed using repeated measures two-way ANCOVA, with post intervention scores as the dependent variable, group as the fixed factor, and baseline values as covariates. The main effect of time was also examined and reported. For non-parametric data (BPI, TSK, SF-36, Stair Climb Test, Trunk Rotation Test, and all postural sway parameters except MF), Quade’s ANCOVA was applied: variables and covariates were ranked, residuals from linear regression were computed, and then analysed via one-way ANCOVA. Effect sizes were reported using partial eta squared (η^2^p). Wilcoxon tests were used for within-group comparisons of non-parametric data. Parametric data were reported as means with 95% confidence intervals, and non-parametric data as medians with interquartile ranges (Q1–Q3). Statistical significance was set at  *p* < 0.05. Analyses were performed using SPSS (v25.0, IBM Corp., Armonk, NY, USA) and GraphPad Prism 10.

## 3. Results

### 3.1. Anthropometrics and Characteristics

At baseline, there were no differences between groups in age (GK, *n* = 12: 58 ± 7 years; PL, *n* = 8: 64 ± 5 years; *p* = 0.238), height (GK 166 ± 6 cm; PL 164 ± 7 cm; *p* = 0.598), weight (GK 66 ± 9 kg; PL 71 ± 10 kg; *p* = 0.248), body mass index (GK 24 ± 3 kg/m 2; PL 26 ± 3 kg/m 2; *p* = 0.062), pain with BPI (GK 4 ± 2; PL 4 ± 1.8; *p* = 0.877), 30 Second Sit To Stand test (GK 14 ± 4; PL 11 ± 2; *p* = 0.129).

### 3.2. Postural Sway

Right leg

Time of single leg stance

A difference between groups in the change over time was observed for the right single leg stance time (Quade’s test: F(1,18) = 5.50, *p* = 0.031, η^2^p = 0.234) with a longer duration in the GK group. Within-group comparisons using Wilcoxon signed-rank tests showed no significant difference for both groups (GK: median 30 s, Q1–Q3 = 30–30, *p* = 0.109, g = 0.643; PL: median 30 s, Q1–Q3 = 22–30, *p* = 0.273, g = −0.559).

Root mean square (RMS)

No differences between groups in change over time were apparent for ML (Quade’s test: F(1,18) = 1.61, *p* = 0.221, η^2^p = 0.082) and AP (F(1,18) = 1.99, *p* = 0.175, η^2^p = 0.100) RMS.

Mean velocity (MV)

A difference between groups in the change over time was apparent for AP MV (Quade’s test: F(1,18) = 4.76, *p* = 0.043, η^2^p = 0.209), whilst ML MV (Quade’s test: F(1,18) = 1.52, *p* = 0.232, η^2^p = 0.078) showed no difference. Within-group analysis using Wilcoxon signed-rank tests revealed a reduction for the GK group for AP MV (median 1.24, Q1–Q3:0.87–1.64, *p* = 0.028, g = −0.572), but not for the PL group (median 1.64, Q1–Q3: 1.51–2.08, *p* = 0.263, g = −0.440). Given the lack of significance for the ML direction, the within-group analysis was not conducted.

Mean frequency (MF)

No significant group × time interactions were found for MF in either ML (F(1,18) = 0.17, *p* = 0.640, η^2^p = 0.013) or AP (F(1,18) = 0.17, *p* = 0.730, η^2^p = 0.007) directions; post hoc tests were therefore not conducted.

**Figure 1:**
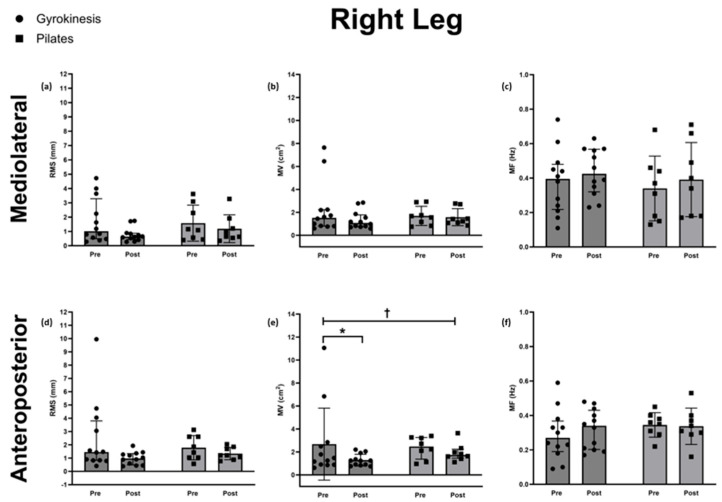
Right leg postural sway parameters, including mediolateral (**a**) root mean square (RMS) (**b**) mean velocity (MV) (**c**) mean frequency (MF) and anteroposterior (**d**) root mean square (RMS) (**e**) mean velocity (MV) (**f**) mean frequency (MF) for the Gyrokinesis group (*n* = 12) and Pilates (*n* = 8) groups before and after the 24-week intervention. Differences between groups in change over time are detonated as † = *p* < 0.05 and within-group differences as * = *p* < 0.05. Data are presented as median ± IQR.

The postural sway parameters for the right leg are shown in Figure 1.

Left leg

Time of single leg stance

A difference between groups in the change over time was detected for the left single leg stance time (Quade’s test: F(1,18) = 11.93, *p* = 0.003, η^2^p = 0.399) with a longer duration in the GK group. Within-group analysis using Wilcoxon signed-rank tests showed no difference for both groups (GK median 30 s, Q1–Q3 = 25–30, *p* = 0.068, g = 0.701; PL median 21 s, Q1–Q3 = 14–30, *p* = 0.068, g = −0.439).

Root mean square (RMS)

There were no differences between groups in change over time for ML (Quade’s test: F(1,18) = 0.523, *p* = 0.480, η^2^p = 0.030) and AP (Quade’s test: F(1,18) = 4.16, *p* = 0.057, η^2^p = 0.197) RMS. Therefore, the post hoc analysis was not conducted.

Mean velocity (MV)

A difference between groups in change over time was found for both ML (Quade’s test: F(1,18) = 6.08, *p* = 0.025, η^2^p = 0.263) and AP (F(1,18) = 5.59, *p* = 0.030, η^2^p = 0.348) MV. Within-group analysis using Wilcoxon signed-rank tests showed a reduction for the GK group in both ML (median 1.25, Q1–Q3: 0.90–1.84, *p* = 0.013, g = −0.700) and AP (median 1.24, Q1–Q3: 0.96–1.62, *p* = 0.008, g = −0.618) MV. No within-group differences were observed in the PL group for ML (median 1.83, Q1–Q3: 1.14–3.12, *p* = 0.249) and AP (median 2.55, Q1–Q3: 1.29–3.91, *p* = 0.128) MV.

Mean frequency (MF)

Mean frequency in AP and ML directions showed no time × group interactions (both *p* > 0.05). Therefore, the post hoc analysis was not conducted.

**Figure 2:**
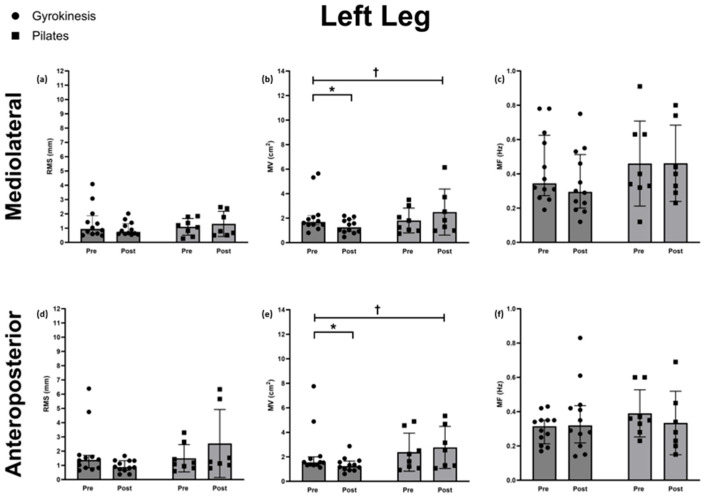
Left leg postural sway parameters, including mediolateral (**a**) root mean square (RMS) (**b**) mean velocity (MV) (**c**) mean frequency (MF) and anteroposterior (**d**) root mean square (RMS) (**e**) mean velocity (MV) (**f**) mean frequency (MF) for the Gyrokinesis group (*n* = 12) and Pilates (*n* = 8) groups before and after the 24-week intervention. Differences between groups in change over time are detonated as † = *p* < 0.05 and within-group differences as * = *p* < 0.05. Data are presented as median ± IQR.

The postural sway parameters for the left leg are shown in Figure 2.

### 3.3. Physical Function/Functional Parameters

A significant group × time interaction was found for the 30-Second Sit-to-Stand test (F(1,18) = 35.152, *p* < 0.001, η^2^p = 0.674). Post hoc analysis revealed a significant increase in the number of sit to stand repetitions in the GK group (*p* < 0.001, g = 2.162), but not the PL group (*p* = 0.879). Similarly, a significant group × time interaction was observed for the TUG test (F(1,18) = 10.574, *p* = 0.005, η^2^p = 0.383), with a significant reduction in test execution time for the GK group (*p* < 0.001, g = −1.214) and no significant change in the PL group (*p* = 0.426). For the Step Test, a significant group × time interaction was observed for both the right and left leg (right: F(1,18) = 18.934, *p* < 0.001, η^2^p = 0.527; left: F(1,18) = 28.246, *p* < 0.001, η^2^p = 0.674). Post hoc comparisons indicated a significant increase in the number of steps in the GK group (both p’s < 0.001, right g = 1.794), but not in the PL group (right: *p* = 0.742; left: *p* = 0.976). A difference between groups in the change over time was observed for the Stair Climb test (Quade’s test: F(1,18) = 5.73, *p* = 0.028, η^2^p = 0.241). Follow-up within-group analyses using the Wilcoxon signed-rank test showed a significant reduction in test execution time in both groups (GK: *p* = 0.002, g = −1.527; PL: *p* = 0.050, g = −0.811). Main effects of time are reported in Table 2, but the focus remains on significant interactions and post hoc comparisons.

### 3.4. Range of Motion and Flexibility

A time × group interaction was observed for the scratch test on both sides (right: F(1,18) = 87.129, *p* < 0.001, η^2^p = 0.837; left: F(1,18) = 150.585, *p* < 0.001, η^2^p = 0.899). Post hoc analysis revealed a significant improvement in the shoulder range of motion on both sides for both groups (GK: right *p* = 0.038, g = −0.136, left *p* < 0.001, g = −0.405; PL: right and left *p* < 0.001, right g = −4.456; left d = −3.709, g = −3.507). No significant interaction was observed for the Sit-and-Reach Test (F(1,18) = 2.68, *p* = 0.120, η^2^p = 0.136), so no post hoc tests were conducted. There was no difference between groups in the change over time for the trunk rotation test on the right (Quade’s test: F(1,18) = 3.58, *p* = 0.075, η^2^p = 0.166) or the left side (Quade’s test: F(1,18) = 3.68, *p* = 0.071, η^2^p = 0.170). Therefore, no further within-group comparisons were performed. Main effects of time were also explored and are reported in Table 2.

### 3.5. Pain

**Figure 3:**
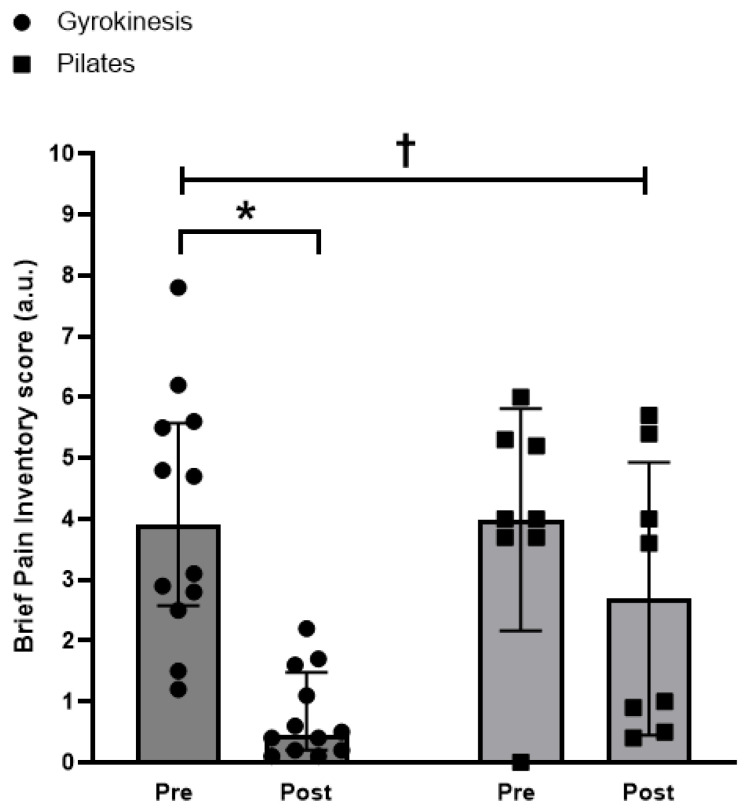
Brief Pain Inventory score for the Gyrokinesis group (*n* = 12) and Pilates (*n* = 8) groups before and after the 24-week intervention. Differences between groups in change over time are detonated as † = *p* < 0.05 and within-group differences as * = *p* < 0.05. Data presented as median ± IQR.

A difference between groups in the change over time was observed in BPI scores, which indicated a significantly greater reduction in the GK group compared to the PL group (Quade’s test: F(1,18) = 5.59, *p* = 0.029, η^2^p = 0.237). Indeed, within-group comparisons using Wilcoxon signed-rank tests showed a significant reduction in perceived pain in the GK group (median 0.45 a.u., Q1–Q3: 0.18–1.5 a.u., *p* = 0.002, g = −2.000), but not in the PL group (median 3.6 a.u., Q1–Q3: 0.9–5.3 a.u., *p* = 0.327) (Figure 3).

### 3.6. Geniophobia

No difference between groups in the change over time was observed for kinesiophobia scores (Quade’s test: F(1,18) = 1.56, *p* = 0.228, η^2^p = 0.080). Therefore, no within group analysis was conducted.

### 3.7. Quality of Life

There were no differences between groups in the change over time for “Physical Health” (Quade’s test: F(1,18) = 1.306, *p* = 0.268, η^2^p = 0.68) or “Mental Health” (Quade’s test: F(1,18) = 3.780, *p* = 0.068, η^2^p = 0.174). Among the individual domains of the SF-36, only “Emotional well-being” showed a significant difference between groups (Quade’s test: F(1,18) = 5.360, *p* = 0.033, η^2^p = 0.229), but within analysis showed no significant changes in both GK and PL groups (*p* > 0.05).

### 3.8. Adherence

Adherence in the GK group was 89% and PL group 92%. Exercise sessions were missed due to lack of time, difficulty commuting, work responsibilities and family commitments. All participants enrolled in the study completed the 24-week intervention. Attendance was recorded for each supervised session. No adverse events were reported during the intervention period.

## 4. Discussion

To our knowledge, this is the first study to compare the effects of the Gyrokinesis method with standard sensorimotor care, represented here by Pilates, in women with KOA. Contrary to our hypothesis, participants in the GK group experienced significantly greater improvements in several clinical parameters. These included greater reductions in perceived pain, superior improvements in postural control, and enhanced outcomes in several functional performance tests.

The reduction in pain observed in both groups supports existing evidence that exercise is an effective non-pharmacological intervention for OA. However, only the GK group demonstrated a statistically and clinically significant decrease in pain (−81% vs. −32%), exceeding established thresholds for a meaningful reduction in pain [36]. This greater analgesic effect may be attributable to the method’s emphasis on three-dimensional, fluid movements, which may reduce joint compression and more effectively redistribute mechanical loads. In this regard, similar modalities, such as Tai Chi and yoga, have demonstrated postural realignment benefits [37], which help mitigate joint stress and decrease nociceptive input [38].

Notably, improvements in postural sway, particularly in single-leg stance time and sway velocity were observed only in the GK group. The concurrent reductions in pain and enhancements in balance suggest that there could be a functional relationship between these two parameters. This hypothesis is supported by recent findings from Alshahrani and Reddy (2023) [39], who demonstrated that in individuals with bilateral KOA, quadriceps weakness is strongly associated with impaired postural stability, and that pain significantly mediates this relationship. The results revealed that reduced quadriceps strength correlated negatively with postural sway variables (r = −0.43 to −0.51, *p* < 0.001), and that individuals with KOA showed markedly greater sway and ellipse area compared to healthy controls. These findings highlight the complex interplay between muscular function, pain, and balance, reinforcing the suggestion that improvements in one domain, such as pain reduction, may facilitate compensatory gains in postural control.

Chronic joint pain in OA is known to disrupt proprioception, induce neuromuscular inhibition, and promote compensatory postural strategies, all of which contribute to increased postural instability [7,38]. In line with this notion, we observed reductions in anteroposterior and mediolateral sway velocities, which may indicate more efficient motor control strategies. It is also important to highlight that reductions in sway velocity occurred without changes in amplitude. These findings are suggestive of superior coordinated corrective actions and improved joint stability [28,40], rather than an increased joint stiffness which can occur when OA becomes more debilitating

While static balance improvements are important, most falls occur during dynamic situations. Crucially, the step test showed improvements in both groups. These observations appear clinically relevant, as postural sway and dynamic balance measures are established predictors of fall risk [41,42], and individuals with OA are susceptible to falls. Thus, improvements in both static and dynamic balance may contribute to greater safety and functional independence in everyday activities.

Functionally, the GK group demonstrated superior improvements compared to the PL group in measures of strength, mobility and coordination (e.g., TUG, Stair Climb, Sit-to-Stand). While both interventions improved upper limb mobility, the GK group led to a wider range of functional benefits. These outcomes may reflect the method’s holistic movement approach, which engages the entire kinetic chain and integrates spinal mobility with extremity control. Future research should focus on characterizing the effects of the GK method from a biomechanical and physiological perspective, for example by examining force distribution, joint mechanical loading and postural alignment following this practice. Additionally, the greater pain reduction observed in the GK group may have increased movement confidence and effort, further supporting improved performance. However, no significant differences were observed in kinesiophobia. Nevertheless, it is plausible that a reduction in pain and improvements in motor control may improve kinesiophobia in other diseased populations or individuals with more severe OA than that used in the current study. In this regard, it is important to consider that we were likely not powered to detect changes in all of our outcome measures, and the small sample size may restrict the generalizability of our findings. Furthermore, while our sample focused exclusively on women, a population highly affected by OA, the efficacy of the GK method for males and different age groups in day-to-day life without supervision remains to be determined.

## 5. Conclusions

In conclusion, the Gyrokinesis method appears more effective than Pilates in improving pain perception, postural control, and physical function in women with KOA. The observed improvements in pain perception and balance suggest that the Gyrokinesis method may not only alleviate OA symptoms but also enhance functional stability and reduce fall risk. These findings support the inclusion of the Gyrokinesis method in multimodal rehabilitation programs and highlight the need for further research to elucidate its biomechanical and neurophysiological mechanisms.

## Figures and Tables

**Table 1:** List of exercises completed in each individual session for the Gyrokinesis group and Pilates group.

Pilates Mat	Gyrokinesis Method
1. Warm-up	1. Arch & Curl series
2. Single leg stretch	2. Spiraling Twists
3. Double leg stretch	3. Sideways Arches
4. Criss-cross	4. Circles/Massaging the Organs
5. Single straight leg	5. Wave Series
6. Roll up	6. Leg/Hip Series
7. Rolling	7. Standing Series
8. Side kick: front/back	8. Cat Back Series
9. Side kick: small circles	9. Curl Back Series
10. Spine twist	10. Alternating Leg Series
11. Rowing 3	11. Scooping Wave Series
12. Rowing 4	12. Pulsation Series
13. Pull straps 1	13. Hip-Knee Mobilization Series
14. Pull straps 2	14. Arches/Back Strengthening
15. Swimming	15. Sphinx Series
16. Teaser 1	16. Abdominal Series
17. Leg pull back	17. Aerobic Class
18. Leg pull front	
19. Mermaid	
20. Rolling down	
21. Cool down	

**Table 2:** Before and after intervention assessments of physical function, range of motion and flexibility for the Gyrokinesis group (*n* = 12) and Pilates (*n* = 8) groups.

	Gyrokinesis (Mean, 95% CI; or Median, Q1–Q3)		Pilates (Mean, 95% CI; or Median, Q1–Q3)		Statistical Significance and Effects Sizes			
Parameter	Week 0	Week 24	Week 0	Week 24	Time × Group	η^2^p (Time × Group)	Time	η^2^p (Time)
30 s Sit to stand (rep)	13.83 (11.3 to 16.3)	24.33 (21.2 to 27.5)	11.29 (9.62–12.95)	11.71 (9.81–13.62)	**<0.001**	0.674	**0.008**	0.342
TUG (s)	5.8633 (5.3 to 6.4)	4.9 (4.5 to 5.3)	7.67 (6.52–8.83)	6.61 (5.89–7.33)	**0.005**	0.383	**0.001**	0.518
Step test right (rep)	14.08 (12.3 to 15.9)	18.9 (17.4 to 20.4)	9.29 (7.90–10.67)	11.14 (9.26–13.03)	**<0.001**	0.527	**<0.001**	0.528
Step test left (rep)	13.58 (11.9 to 15.3)	19.2 (17.9 to 20.6)	9.43 (7.84–11.02)	11.14 (9.11–13.17)	**<0.001**	0.674	**<0.001**	0.592
Stair Climb (s)	9 (8–9.9)	7.1 (6.2–7.6)	12.7 (11.5–12.9)	9.3 (8.8–9.7)	**0.028**	0.241	n/a	n/a
Scratch test right (cm)	23.750 (20.1 to 27.3)	23 (19.8 to 26.1)	30.00 (25.28–34.72)	11.79 (9.79–13.78)	**<0.001**	0.837	0.437	0.036
Scratch test left (cm)	28 (23.6 to 32.3)	25.5 (22.6 to 28.5)	33.43 (27.25–39.61)	14.21 (10.33–18.10)	**<0.001**	0.899	0.387	0.044
Sit and reach (cm)	2.2 (−3.8 to 8.2)	7.7 (3.0 to 12.3)	−2.00 (−9.18–5.18)	2.29 (−2.94–7.51)	0.120	0.136	**0.001**	0.512
Trunk rotation right (°)	37.5 (29.4–41.9)	50.7 (41.2–57.5)	37.9 (30.6–42.3)	40.1 (38.9–42.7)	0.075	0.166	n/a	n/a
Trunk rotation left (°)	31.4 (27.5–39.1)	49.7 (39.7–54.5)	36.3 (32.4–39.3)	42.7 (36.3–43.9)	0.071	0.170	n/a	n/a

## Data Availability

The data is available upon request.

## Abbreviations

The following abbreviations are used in this manuscript:

η^2^p	Partial eta square
AP	Antero-posterior
BPI	Brief pain inventory
CI	Confidence interval
ES	Effect size
GK	Gyrokinesis method
ICC	Intraclass correlation coefficient
KOA	Knee osteoarthritis
MF	Mean frequency
ML	Medio-lateral
MV	Mean velocity
OA	Osteoarthritis
PL	Pilates
QoL	Quality of life
RMS	Root mean square
ROM	Range of motion
SF-36	36-item short form survey
TSK	Tampa scale of kinesiophobia
TUG	Time up and go
